# Negative Self-Disclosure on the Web: The Role of Guilt Relief

**DOI:** 10.3389/fpsyg.2017.01068

**Published:** 2017-06-28

**Authors:** Liat Levontin, Elad Yom-Tov

**Affiliations:** ^1^Faculty of Industrial Engineering and Management, Technion – Israel Institute of TechnologyHaifa, Israel; ^2^Microsoft Israel R&D CenterHerzliya, Israel

**Keywords:** self-disclosure, guilt, guilt relief, prosocial behavior, online forums

## Abstract

In this paper, we suggest people use anonymous online forums as platforms for self-disclosing actions they feel guilty about—such as transgressions and unethical behaviors—with the goal of achieving guilt relief through others’ reactions. We support this proposition by analyzing field data extracted from Yahoo Answers, an online question-and-answer website. Our analysis shows the level of guilt relief an answer is expected to offer the “asker” (the self-disclosing person) is positively associated with the asker’s likelihood of selecting that answer as the “best” response to the self-disclosure. Furthermore, following receipt of a guilt-relieving answer, an asker becomes less likely to engage in prosocial behavior, which is another type of guilt-relieving action.

## Introduction

Facebook, Twitter, Community Question Answering (CQA) sites (e.g., YahooAnswers), and review sites such as TripAdvisor, Yelp, or Booking.com that include reviews of hotels, restaurants, and any other commodity are so appealing partly because they include people’s self-disclosures. Self-disclosure refers to the degree to which an individual shares personal information with others (Altman and Taylor, 1973). Individuals seem to self-disclose everything: their love and lovers, their happiest events, their illnesses and troubles, their trips and vacations, their political views, their children’ achievements and great moments, and more.

In the current research, we explore the effect of negative self-disclosure on consequent prosocial behavior. We suggest that although online self-disclosure has become an everyday activity for most people (sharing something on Facebook, writing a review, etc.), many people are still reluctant to self-disclose their negative behaviors, such as unethical behaviors. One of the main motivators for disclosures of negative personal information, those that elevate feelings of guilt, is to achieve guilt relief. We thus propose that when people disclose their wrongdoings, they expect to obtain guilt relief. If achieved, they require no further relief, and are less likely to engage in other means of guilt relief, such as prosocial behavior.

### Self-Disclosure Regarding Negative Events

Self-disclosure is an important factor in interpersonal relationships and is a crucial part of relationship development (Altman and Taylor, 1973; Derlega et al., 1993). In fact, intimate relationships are built through a process of reciprocity of self-disclosure, and people who engage in intimate self-disclosures tend to be liked more than people who disclose less (Collins and Miller, 1994). Research has demonstrated the importance of self-disclosure in many social contexts. Thompson (1991) found it advances negotiations: the disclosing of information improved the accuracy of negotiators’ judgments about the other party and lead to more mutually beneficial, integrative negotiation agreements. Of note, joint outcomes improved significantly even when only one member of the bargaining pair provided information. Stasser et al. (1995) found group decision-making could benefit from self-disclosure that enables the use of members’ unique knowledge, and that the group may fail to benefit if information only one group member holds is not disclosed and is omitted from discussion. In the work place, mentors’ self-disclosure helps build core capabilities of the organization that include norms, values, and employees’ critical skills (Swap et al., 2001), and find the exchange of privacy-related personal information for customized benefit offerings attractive (White, 2004).

Notably, however, people disclose significantly more personal information, and disclose it more quickly, when interacting with strangers than when interacting with acquaintances (e.g., John et al., 2011). Similarly, willingness to disclose information is significantly higher in the context of computer-mediated communication than in face-to-face-settings (e.g., Joinson, 2001; Bargh et al., 2002).

Negative self-disclosure, sharing negative personal information with others, is a common ritual rooted in most main religions and cultures (e.g., Christianity, Islam, Judaism, and Buddhism), where it serves as a means of cleansing the individual’s soul (Kassin and Gudjonsson, 2004). Similarly, most modern mental health treatments and social support groups are based on the premise that disclosure of one’s problems, traumas, and transgressions holds the power to heal. Indeed, disclosure has been shown to have positive effects on both psychological and physical markers (Pennebaker, 1997, 2012).

Although religions and societal norms expect and encourage negative self-disclosures, people who feel guilty about their misbehavior often have various reasons to feel reluctant to self-disclose their wrongdoings. People are likely to experience vulnerability when they reveal personal information, particularly their innermost attitudes and emotions, including their feelings of guilt (e.g., Lehman et al., 1986; Derlega et al., 1993). Indeed, self-disclosure of negative information (e.g., sins or wrongdoings) may lead to less liking by others (Forest and Wood, 2012), and people have reservations about self-disclosing negative and embarrassing information. For example, many clients report that during their mental health intake, the first meeting with a therapist, they did not provide their therapist with important information, including histories of their chief complaints (Barry et al., 2000). Surveys of criminal confessions (guilt-related self-disclosures) in the United Kingdom estimated that confession rates range from 55 to 62% across various studies and settings (Pearse et al., 1998). A more recent study of confessions by non-criminals also found low confession rates; some participants even restricted their confessions by admitting to some, but not all, of their unethical behavior (Peer et al., 2014). Even consumers who find self-disclosure of privacy-related personal information for customized benefit offerings (relative to non-customized offerings) attractive find the exchange of customized offerings for embarrassing information unattractive (White, 2004).

As such, negative self-disclosure usually takes place in a unidirectional manner, wherein the self-disclosing person presents his or her inner feelings to an unresponsive audience such as a priest, a therapist, a page, or strangers on the internet (Pennebaker et al., 1987; Kassin and Gudjonsson, 2004; Misoch, 2015).

We suggest, however, that in some cases, the discloser’s main goal is to obtain others’ reactions, as may be the case in disclosure regarding negative events (e.g., transgressions, unethical behaviors) that the individual feels guilty about, that is, perceives himself or herself as having caused (Neumann, 2000), and aims to achieve guilt relief. Indeed, feelings of guilt and the desire to experience a sense of relief are among the factors that motivate people to make confessions (Gudjonsson and Sigurdsson, 1999).

Guilt is an unpleasant emotion that is aroused when the actor causes, anticipates causing, or is associated with an aversive event such as committing an unethical behavior (Zeelenberg and Breugelmans, 2008; Cohen et al., 2011; de Hooge et al., 2011). Guilt is an undesirable emotion, at least in more individualistic cultures (Tamir, 2016); thus, when people experience guilt, their common response is to seek means of relieving it (Baumeister et al., 1994).

Guilt relief is important to people because guilt feelings lead to a sense of resource deficiency. Specifically, guilt can detract from core resources such as sense of pride, a sense that life is peaceful, the feeling that one knows who she is, and a general positive feeling about oneself (Hobfoll, 2001), and people go to great lengths to protect themselves from a lack of resources (Levontin et al., 2015). Notably, however, resource depletion diminishes feeling of guilt (Xu et al., 2012).

Yet one can relieve guilt in ways other than through self-disclosure, for example, by seeking justification for misbehavior, showing willingness to take reparative actions toward those harmed (Rotella and Richeson, 2013), experiencing pain (Bastian et al., 2011), and engaging in prosocial behavior (Andreoni, 1990).

### Guilt and Prosocial Behavior

Prosocial behavior represents a broad category of acts that are defined by some significant segment of society and/or one’s social group as generally beneficial to other people (Penner et al., 2005). People help others for many reasons. One mechanism influencing helping others is reciprocal altruism that suggests helping is based on the probability of being helped in return (Penner et al., 2005). The norm of reciprocity apparently exists in many cultures (Schroeder et al., 1995), and people are more likely to help those who offer help (Boster et al., 2001). Helping can also be the result of complying with social norms, complying with internalized personal norms, or avoiding guilt (Batson and Shaw, 1991).

Research evidence connects prosocial behavior with feelings of guilt (Andreoni, 1990; Basil et al., 2008), and experimentally induced feelings of guilt increased individuals’ willingness to engage in prosocial behaviors (Cunningham et al., 1980; Gino and Pierce, 2009; Cryder et al., 2012; Xu et al., 2012), even when guilt was non-consciously induced (Zemack-Rugar et al., 2007). In social-dilemma games, people acted more prosocially after an autobiographical recall procedure induced feelings of guilt, or after they made an unfair offer in an earlier round of the game. An induction of guilt increased prosocial behavior, whereas an induction of fear did not (Ketelaar and Au, 2003; de Hooge et al., 2007; Nelissen and Dijker, 2007).

### The Current Research

We propose that people self-disclose their intimate perceived wrongdoings, those that elevate feelings of guilt, to achieve guilt relief through others’ reactions. We explore the relationship between self-disclosure and guilt relief by analyzing field data regarding people’s intimate negative self-disclosures in an online question-and-answer forum. Such forums offer anonymity, which decreases the risk of being rejected. They also are not unidirectional, and therefore allow for true responses from the audience (Pelleg et al., 2012). Thus, such forums allow people to self-disclose their intimate perceived wrongdoings, with the goal of achieving guilt relief, and can serve as an alternative to a therapist or priest.

We further propose that when people disclose their wrongdoing and subsequently obtain guilt relief through others’ reactions, they require no further relief, and are therefore less likely to engage in other means of guilt relief, such as prosocial behavior. Our data enable us to examine this hypothesis as well.

## Materials and Methods

### Dataset

**FIGURE 1:**
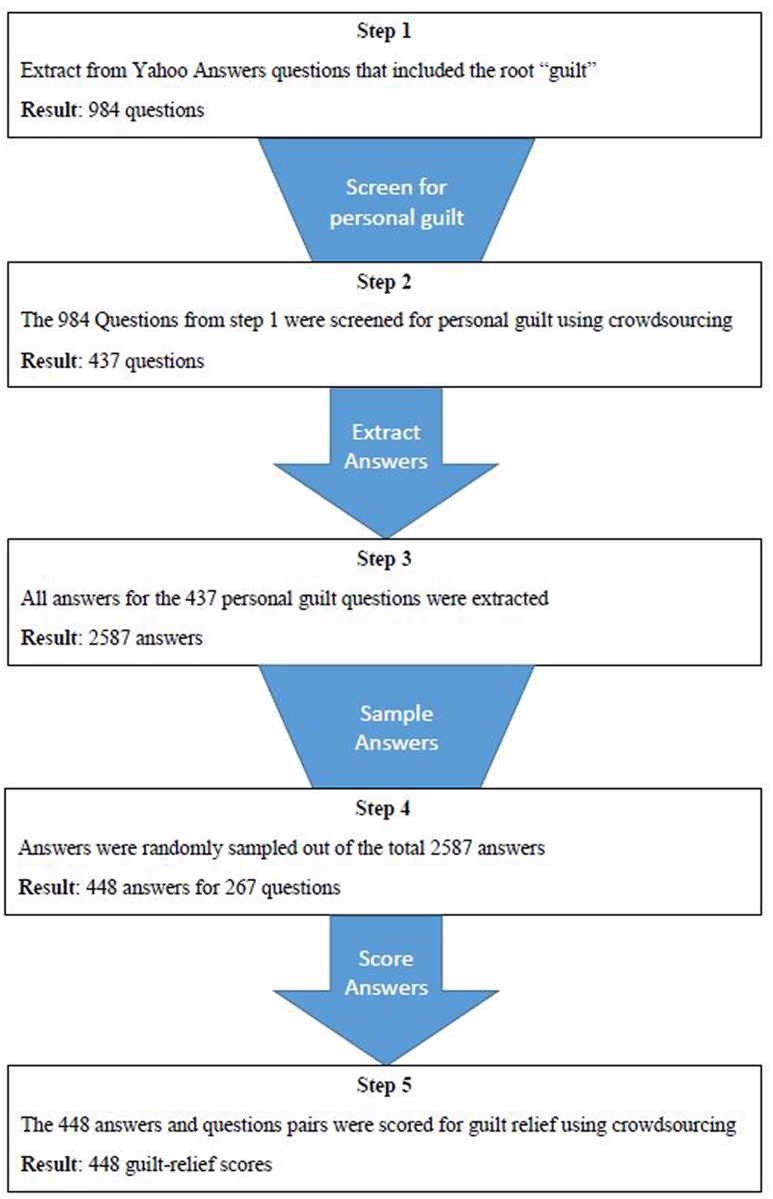
A flow chart of the steps in data set preparation.

We first searched the online question-and-answer website Yahoo Answers^1^ for questions that included the root “guilt” (the words “guilt” and “guilty”; see **Figure 1**). We extracted 984 such questions. Next, we excluded questions that focused on non-personal guilt (**Figure 1**). To identify these questions, we submitted the 984 questions to independent judges from CrowdFlower, a crowdsourcing service. Five judges labeled each question according to whether or not it described personal guilt. For each question, the majority of judges were in agreement regarding the label of the question, and for 79% of the questions, four or more of the labelers were in agreement. An example of a question that was excluded from the data because the root “guilt” did not reflect a feeling of personal guilt asked, “[Why isn’t God] powerful enough to have removed evil people while sparing babies… too young to be guilty of a sin?” We ended up with 437 questions that included feelings of personal guilt and could be referred to as negative self-disclosures (**Figure 1**). A sample question reads, “Ladies or gentlemen have you ever while on a diet ever fallen of the wagon like eating something you shouldn’t of? I just eaten 4 chocolate digestives and now I feel guilty I have recently started going to the gym so thank god I can burn it off but I still feel guilty though anyone been the same?” Each question in our negative self-disclosure data set received between 1 and 50 answers from users of the Yahoo Answers forum (*M* = 8.80*, SD* = 8.50); our data thus included 2,587 answers (**Figure 1**). From this data set, we randomly chose 448 answers (corresponding to 267 questions; 1.7 answers per question on average, *SD* = 1.40), slightly more than 15% of the data points (**Figure 1**).

### Measures

#### Guilt-Relief Score

Each of the 448 question–answer pairs in our data set was rated by five CrowdFlower judges for how guilt relieving the answer was, on a seven-point scale (1 = *Not at all*, 7 = *Very much*). One hundred and ten CrowdFlower participants evaluated, on average, 23 items each. We averaged the five ratings for each pair to receive a guilt-relief score. Scores ranged between 1.2 and 6.8 (*M* = 4.53, *SD* = 1.06).

#### Best Answer (Selected by Asker)

On Yahoo Answers, an “asker” (in our case, the user who has posted a negative self-disclosure) has the option to choose the “best answer” for his or her question out of all the answers received. We coded each answer such that 1 reflected an answer the asker chose as the best response to the negative self-disclosure. Askers chose 55 (12.3%) of the answers in our data set as best answers. We expected that answers chosen as best answers would be more guilt relieving than other answers.

#### Thumbs-Up/Down Votes

All users of Yahoo Answers have the option to “vote” that they liked (disliked) a given answer by clicking on a thumbs-up (thumbs-down) button next to that answer. Each answer in our data set received between 0 and 8 thumbs-up votes (*M* = 0.62, *SD* = 1.17). The most frequent result was zero (*N* = 291, 65%), followed by one thumbs-up vote (*N* = 96, 21.4%). Each answer also received between 0 and 9 thumbs-down votes (*M* = 0.40, *SD* = 0.94). The most frequent result was zero (*N* = 343, 76.6%), followed by one thumbs-down vote (*N* = 59, 13.2%). If the asker does not mark an answer as the “best answer,” the answer that received the most thumbs-up votes (minus thumbs-down votes) is marked as the “best” according to the community of users. Seventy-two of the answers in our data set (16.1%) were selected as best answers according to the community of users. Given the many criteria, aside from guilt relief, that are likely to lead users to vote for a given answer, we do not expect the answers the community rated as best answers to be more guilt relieving than other answers.

#### Order of Answer

We coded each answer according to the position in which the answer appeared within the sequence of all answers provided to the corresponding question. Specifically, 0 reflected the first answer or reaction to the negative self-disclosure, 1 reflected the second answer or reaction, and so on. The value of this order variable ranged between 0 (*N* = 67, 15%) and 45 (*M* = 6.77, *SD* = 8.27).

#### Time between Question and Answer

We coded the amount of time that passed between the posting of the negative self-disclosure and the posting of the corresponding answer in the pair (in minutes, *M* = 3.50, *SD* = 71.84).

#### Time from Answer to Asker’s First Reaction to Others

For each pair of a negative self-disclosure and an answer, we first coded the amount of time that had passed between the posting of the negative self-disclosure and the asker’s first reaction to another person’s question on the site. From this value, we subtracted the amount of time that had passed between the posting of the negative self-disclosure and the posting of the answer in the pair. Thus, we obtained the time between the asker’s receipt of a given answer (reaction) and his or her first reaction to others. Note this measure can also receive negative values, because an asker can react to another user’s question before receiving a specific answer to his or her negative self-disclosure. Indeed, the value of this measure ranged between 1520.65 and 2251.06 minutes (*M* = 11.79, *SD* = 132.60). We expected that, following guilt relief, askers would behave *less* prosocially and would be *less* inclined to help others by answering their questions.

We suggest the goal of self-disclosure of one’s guilt-evoking behaviors is to achieve guilt relief. Yet the responses one receives to a negative self-disclosure may not relieve that guilt; in fact, some answers might achieve the opposite and serve as guilt reminders. For example, one of the askers in our data set posted the following negative self-disclosure:

I feel a bit guilty: I’m 18 weeks pregnant, and overall eat very healthy. The only drinks I consume are water, milk, juicy juice, and crystal lite. I do, however, have about 2–3 green tea Frappuccinos a week from Starbucks. I don’t know how much caffeine are in those, nor do I want to. I feel a bit guilty drinking these, but I’m seriously addicted, have been since before I was pregnant. Is it unhealthy for my baby to be drinking a lot of these?

Our data set includes two answers to this negative self-disclosure:

(1) “It shouldn’t do too much to the baby as long as your healthy” (was not chosen as the best answer by the asker; received 5 thumbs-up votes; guilt-relief score of 5.4 out of 7).
(2) “Yes I use 2 work at Starbucks they r very bad 4 u” (was not chosen as the best answer by the asker; received 0 thumbs-up votes; guilt-relief score of 2.8 out of 7).

Answering other people’s questions and providing informal counsel is a prosocial act of help-giving directed toward individuals (Brief and Motowidlo, 1986). Disclosures of negative personal information, like all other Yahoo Answers community members, can behave prosocially by giving answers to other people’s questions on the site. Those who help and answer questions on site receive some benefits: they can be thanked by the asker who can choose this answer as the best answer, by other people from the community that can give a “thumbs-up” rating to the answer, and by the site itself, which gives people two points for every answer. However, we suggest that disclosures of negative personal information are interested in guilt relief more than anything else. As such, when guilt relief is achieved, their prosocial tendencies to answer other people’s questions will decrease.

We used our data to test the following operational hypotheses:

H1: An answer’s guilt-relief score will be positively related to the likelihood of the asker choosing that answer as the best answer to the negative self-disclosure.

H2: An answer’s guilt-relief score will be negatively related to the asker’s likelihood of subsequently helping other askers by answering their questions.

## Results

To test the suggestion that guilt relief is the motivation for self-disclosure about guilt-provoking behavior, we first used logistic regression to test the relation between the guilt-relief score and an asker choosing an answer as the best answer. Askers’ choices of best answer served as the DV (1 = best answer, 0 = not best answer). The predictors were total number of answers, guilt-relief score, order of the answer, number of thumbs-up votes, and all two-way interactions (**Table 1**). Results suggest that, controlling for the total number of answers, the number of thumbs-up votes, and the order of the answer, a one-unit increase in the guilt-relief score increases by 60% the odds of an answer being chosen as the best answer.

**Table 1:** Results of logistic regression on the asker’s likelihood of choosing the answer as the best answer.

	Model 1			Model 2			Model 3		
Variable	*B*	*SE*	*OR*	*B*	*SE*	*OR*	*B*	*SE*	*OR*
Constant	-3.82^∗∗∗^	0.78	0.02	-4.46^∗∗∗^	0.90	0.01	-3.49^∗∗^	1.06	0.03
Total number of answers	-0.01	0.01	0.99	-0.01	0.18	0.99	-0.01	0.01	0.99
Guilt-relief score	0.44^∗∗^	0.15	1.55	0.56^∗∗∗^	0.18	1.76	0.47^∗^	0.21	1.60
Thumbs-up votes				0.93	0.65	2.54	0.84	0.68	2.32
Guilt relief × Thumbs-up				-0.20	0.13	0.82	-0.20	0.14	0.82
Order of answer							-0.16	0.17	0.85
Guilt-relief score × order							0.01	0.03	0.94
Thumbs-up votes × order							0.02	0.01	0.99
-2 Log likelihood	323.79			321.41			306.72		
χ^2^	9.89, *df* = 2, *p* = 0.007			12.27, *df* = 4, *p* = 0.015			26.96, *df* = 7, *p* = 0.000		

*OR = Odds Ratio (ExpB). ^∗^p < 0.05; ^∗∗^p < 0.01; ^∗∗∗^p < 0.001.*

To test the alternative explanation that guilt-relieving answers are simply better answers, we ran another logistic regression with the community’s choice of “best answer” as the DV (1 = best answer, 0 = not best answer); the predictors were the total number of answers, the guilt-relief score, the order of the answer, and their interaction. Indeed, none of these predictors reached significance, and importantly, the guilt-relief score did not predict the community’s best-answer choices (**Table 2**).

**Table 2:** Results of logistic regression on number of thumbs-up votes.

	Model 1			Model 2		
Variable	*B*	*SE*	*OR*	*B*	*SE*	*OR*
Constant	-1.92^∗∗^	0.60	0.15	-1.45	0.80	0.23
Total number of answers	0.01	0.01	1.01	0.01	0.01	1.01
Guilt-relief score	0.04	0.12	1.04	0.12	0.17	1.13
Order of answer				0.03	0.17	1.03
Guilt-relief score × order				-0.06	0.04	0.94
-2 Log likelihood	394.59			343.70		
χ^2^	0.42, *df* = 2, *p* = 0.812			51.30, *df* = 4, *p* = 0.000		

*OR = Odds Ratio (ExpB). ^∗^p < 0.05; ^∗∗^p < 0.01; ^∗∗∗^p < 0.001.*

Next, we examined the relationship between guilt relief and prosocial behavior, as reflected in the likelihood of helping other askers by giving answers to their questions at the time between the negative self-disclosure and the recipient of a guilt-relieving (or not) answer. We used a Cox proportional hazards regression model to estimate this relationship. Specifically, for each answer A to a negative self-disclosure D, the dependent variable was the time that passed between receiving answer A and giving an answer to another person’s question. If the asker did not answer any questions on the site (*N* = 65), or if the asker answered another person’s question before receiving answer A to his/her negative self-disclosure D (*N* = 146), we treated the observation as censored. The data thus included *N* = 237 events and *N* = 211 censored cases. The predictor was answer A’s guilt-relief score, controlling for whether the asker chose answer A as the best answer. That, because the worm glow from receiving an answer perceived as the best answer can positively influence prosocial behavior, in the form of helping others, beyond the answers’ guilt relief effect. The estimated hazard ratio of the guilt-relief score was exp(B) = 0.89 (*B* = -0.12, *SE* = 0.06, *p* = 0.049). We repeated the analysis without controlling for whether the asker chose answer A as the best answer, and received similar but only marginally significant results: the estimated hazard ratio of the guilt-relief score was exp(B) = 0.90 (*B* = -0.11, *SE* = 0.06, *p* = 0.077). This result indicates that, as hypothesized, when an asker receives a more guilt-relieving answer, he or she becomes less likely to subsequently help others by answering their questions.

## Discussion

Analyzing data from Yahoo Answers, we have shown the guilt-relief score corresponding to a given answer predicts the likelihood that the asker will choose that answer as the “best answer” to his or her negative self-disclosure. However, this score does not predict the likelihood that the community will choose an answer as being the “best”—ruling out the alternative explanation that guilt-relieving answers are inherently “better” than other answers. Furthermore, an asker who has received a guilt-relieving answer subsequently becomes less likely to engage in prosocial behavior (answering others’ questions), which is another type of guilt-relieving action (Gino and Pierce, 2009).

Prosocial behaviors are positive social acts carried out to produce and maintain the well-being of others and the greater good of society. Prosocial behavior, kindness, generosity, and cooperation are in many ways the glue that holds the social fabric together. Therefore, the fact that managers, educators, institutions, and religions encourage prosocial behaviors is unsurprising (e.g., Brief and Motowidlo, 1986; Kidron and Fleischman, 2006; Hyson and Taylor, 2011). As such, self-disclosure, a ritual rooted in most main religions and cultures, should not negatively influence prosocial behaviors that smooth our social interactions. However, the current research shows it sometimes does: when self-disclosures relieve guilt, subsequent prosocial behaviors decrease.

Research suggests people tend not to adopt stable disclosure strategies; rather, they base their disclosure decisions on transient cues except for one strategy—that people self-disclose less in the face of cognitive disfluency than in the face of cognitive fluency (Alter and Oppenheimer, 2009). Accordingly, identifying factors that motivate self-disclosure is practically and theoretically important. The current work points to a factor that may motivate self-disclosure: achieving guilt relief. Specifically, we have observed that people who use online forums to disclose guilt feelings tend to prefer responses that relieve their guilt, and upon receiving such responses, they subsequently become less likely to engage in other guilt-relieving actions (prosocial behavior).

Our studies have several limitations. First, the search we used for the terms “guilt” or “guilty” in people’s questions leaves out linguistic indicators of guilt that aren’t necessarily captured by the word “guilt.” Future research should examine people’s behavior following a guilt-relieving answer for negative self-disclosures regardless of the use of the root “guilt” in the question. One possible hypothesis is that the prosocial behavior of self-disclosures’ of negative behaviors that do not use the root “guilt” in their question will be less affected by a guilt-relieving answer, because guilt relief is not the asker’s main goal. However, another possible hypothesis is that those who self-disclose a negative event and do not use the root “guilt” in their question are those who feel guilty but are trying to hide it, and thus a guilt-relieving answer will affect their behavior even more.

Second, our measure of prosocial behavior—answering other’s questions on Yahoo Answers—is an imperfect and rather narrow measure of prosocial behavior. Askers might engage in other forms of prosocial behavior when they feel guilty, and these other prosocial behaviors are less or more affected by guilt-relieving answers than answering others’ questions on Yahoo Answers. However, answering others’ questions is a measure of askers’ actual prosocial behavior in a natural setting.

Another limitation of this study, and of field studies in general, is that we didn’t have access to askers’ mental states and thus could only infer them. We inferred guilty feelings of askers from their use of the root “guilt” in the question and after judges labeled each question according to whether or not it described personal guilt. However, we cannot infer askers’ perceptions of the severity of their transgressions. Hypotheses about the relation between the askers’ and responders’ perceptions of the severity of the transgressions and how guilt relieving the answers were should be tested in future research. Next, we inferred the guilt relief of askers by using judges’ ratings for the level of guilt relief in the answers. We showed that when judges rated an answer as guilt relieving, the askers tended to pick it as the best answer. However, this answer did not necessarily serve the psychological function of guilt relief. Best answers could have additional diagnostic information or advice that warrants its choice as a top answer. Future research should analyze best answers of negative self-disclosures to better understand their overall function.

These limitations notwithstanding, our data set represents a unique observation of prosocial behavior in the real world. As such, it allows researchers to observe a social phenomenon that has thus far been studied mostly under controlled conditions.

The current research opens several avenues for future research. First, future research could focus on different occasions where self-disclosures occur to test the effect of self-disclosure on guilt relief and subsequent prosocial behavior. Confession (Kassin and Gudjonsson, 2004) is one occasion that encourages self-disclosure, and mental health treatment or participation in a social support group (Pennebaker, 1997, 2012) is another. Whereas self-disclosures on the web may relieve the guilt that follows transgressions, a confession to a religion representative (God, priest, etc.) may serve more as a guilt reminder and encourage prosocial behavior to relieve the guilt. Second, future research should establish the causality effect of self-disclosure on subsequent prosocial behavior as mediated by guilt.

Given the importance of negative self-disclosures for the monitoring of the community, improving its health, and even crime control (Kassin and Gudjonsson, 2004), offering options of guilt relief may prove beneficial in encouraging negative self-disclosures. Online forums could play a significant role in achieving this goal. People disclose significantly more personal information, and disclose it more quickly, when interacting with strangers than when interacting with acquaintances (e.g., John et al., 2011). Similarly, willingness to disclose information is significantly higher in the context of computer-mediated communication than in face-to-face-settings (e.g., Joinson, 2001; Bargh et al., 2002). We are only on the verge of understanding the potential of online environments to promote the achievement of such goals.

## Author Contributions

LL and EY-T developed the study concept and contributed to the study design. Data collection was performed by EY-T. LL performed the data analysis and interpretation and drafted the manuscript, and EY-T provided critical revisions. Both authors approved the final version of the manuscript for submission.

## Conflict of Interest Statement

EY-T, declares an affiliation with Microsoft Research Israel. The other author declares that the research was conducted in the absence of any commercial or financial relationships that could be construed as a potential conflict of interest.

## Footnotes

1 https://answers.yahoo.com
